# *Leptospira interrogans* Stably Infects Zebrafish Embryos, Altering Phagocyte Behavior and Homing to Specific Tissues

**DOI:** 10.1371/journal.pntd.0000463

**Published:** 2009-06-23

**Authors:** J. Muse Davis, David A. Haake, Lalita Ramakrishnan

**Affiliations:** 1 Immunology and Molecular Pathogenesis Graduate Program, Emory University, Atlanta, Georgia, United States of America; 2 Veterans Affairs Greater Los Angeles Healthcare System, Los Angeles, California, United States of America; 3 Departments of Medicine and Urology, David Geffen School of Medicine at UCLA, Los Angeles, California, United States of America; 4 Departments of Microbiology, Medicine and Immunology, University of Washington, Seattle, Washington, United States of America; Institut Pasteur, France

## Abstract

Leptospirosis is an extremely widespread zoonotic infection with outcomes ranging from subclinical infection to fatal Weil's syndrome. Despite the global impact of the disease, key aspects of its pathogenesis remain unclear. To examine in detail the earliest steps in the host response to leptospires, we used fluorescently labelled *Leptospira interrogans* serovar Copenhageni to infect 30 hour post fertilization zebrafish embryos by either the caudal vein or hindbrain ventricle. These embryos have functional innate immunity but have not yet developed an adaptive immune system. Furthermore, they are optically transparent, allowing direct visualization of host–pathogen interactions from the moment of infection. We observed rapid uptake of leptospires by phagocytes, followed by persistent, intracellular infection over the first 48 hours. Phagocytosis of leptospires occasionally resulted in formation of large cellular vesicles consistent with apoptotic bodies. By 24 hours, clusters of infected phagocytes were accumulating lateral to the dorsal artery, presumably in early hematopoietic tissue. Our observations suggest that phagocytosis may be a key defense mechanism in the early stages of leptospirosis, and that phagocytic cells play roles in immunopathogenesis and likely in the dissemination of leptospires to specific target tissues.

## Author Summary

Leptospirosis is a common bacterial infection in many tropical regions of the world that causes serious and often fatal disease in humans. The infection is transmitted by carrier animals, especially rats and other rodents, that release the leptospire bacteria from their kidneys into their urine. Humans are infected through exposure of broken skin or mucous membranes to contaminated water. Little is known about how or why the bacteria traffic from these sites specifically to the kidneys. The zebrafish embryo is a popular model organism for studying embryonic development, in part because of the ease with which living cells within the transparent embryos can be studied under the microscope. In this study, we use leptospire-infected zebrafish embryos to examine early leptospirosis by microscopy. In the first days of infection, the embryos appear normal. We find that leptospires are readily ingested (but not killed) by white blood cells called phagocytes. Later, infected cells are found specifically in a tissue near the dorsal aorta. This site may be a tissue that produces new blood cells and may represent a conduit for subsequent tissue targeting of the organisms. Our findings suggest that the zebrafish model may be useful for studying the pathogenesis of leptospirosis.

## Introduction

Though traditionally thought of as a tropical disease, leptospirosis is endemic worldwide due to widespread infection of urban and sylvatic rodents and other animal reservoir hosts. In areas of the world with high levels of rodent exposure, human infection is common and frequently progresses to serious disease or death [1],[2]. Although much has been learned about the biology and transmission of *Leptospira* species, the mechanisms of their pathogenesis and host colonization remain largely unknown. Leptospires colonize the renal tubules of reservoir hosts, from where they are shed in the urine and infect new hosts via mucosal surfaces and abraded skin. In the reservoir host, there is transient low-level hematogenous dissemination, followed by chronic infection limited to the kidney [1],[3],[4]. In contrast, susceptible hosts experience a heavy burden of infection in the bloodstream and multiple organs. The eventual antibody response precipitates an intense inflammatory reaction associated with hepatorenal failure. A key difference between reservoir and susceptible hosts is the ability of the TLR4 innate immune receptor to recognize leptospiral lipopolysaccharide (LPS) [5],[6]. Murine peritoneal macrophages are strongly stimulated by purified leptospiral LPS, while human macrophages are unable to respond to leptospiral LPS via the TLR4 pathway [5]. Taken together, these studies suggest that early containment of infection via innate mechanisms, including recognition of leptospiral antigens and phagocytosis by macrophages, is essential for effective immune defense [7]. Previous in vitro studies have demonstrated that macrophages are capable of phagocytosing leptospires [8],[9].

A variety of animal models of leptospirosis have been established, each with unique advantages and drawbacks. Guinea pigs [10] and hamsters [11],[12] are the primary models of hosts susceptible to acute disease, while several animals including mice [4], rats [3], monkeys [13], dogs [14] and skunks [15] can be experimentally infected and seem variously plausible as models of reservoir hosts. It is not certain to what degree these various model hosts retain features of natural infection and colonization.

**Figure 1:**
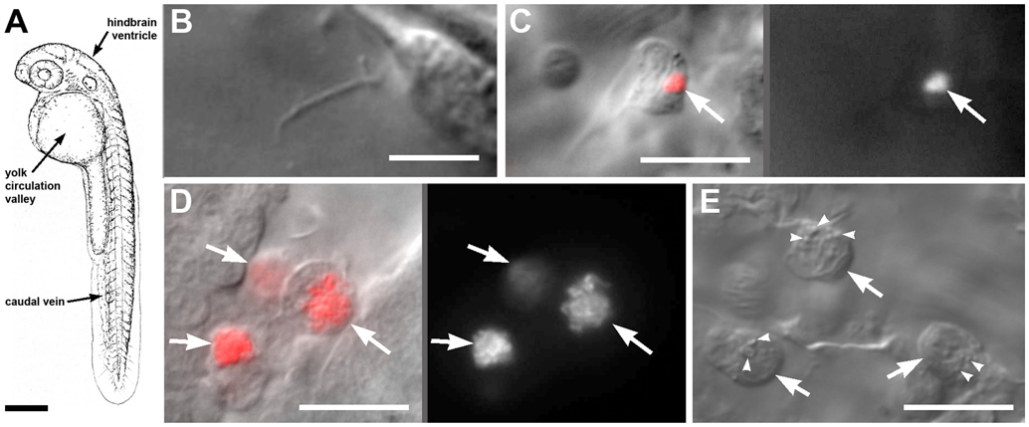
Cellular details of early phagocyte-leptospire interactions. A. Schematic view of 36hpf zebrafish embryo, with injection and observation sites indicted. Scale bar, 300 µm. B. Phagocyte containing leptrospires within two hours of intravenous infection. Left, DIC overlay; right, SYTO 83 fluorescence. C. Single leptospire visible by DIC microscopy shortly after injection. See also Video S1. D. Phagocytes containing large numbers of leptospires four hours after injection into hindbrain ventricle. Left, DIC overlay; right, SYTO 83 fluorescence. E. Phagocytes (arrows) containing leptospires also acquired numerous small cytoplasmic vesicles (arrowheads). See Video S2. All scale bars 20 µm unless noted otherwise.

The zebrafish is increasingly used as a model organism for bacterial pathogenesis, with published studies of adult infection with pathogens including mycobacteria [16], streptococci [17], and Edwarsiella [18]. The ability to conduct forward genetic screens, along with the economy of infecting large numbers of animals are key advantages to this model [19],[20]. Beyond these, the zebrafish embryo allows unparalleled in vivo microscopy and tracking of host-pathogen interactions involving fluorescently labeled bacteria. Minute details of the early steps of bacterial pathogenesis have been published using zebrafish embryos infected with *Mycobacterium marinum* [21],[22],[23], *Salmonella enterica* [22],[24] and *Pseudomonas aeruginosa* [25]. By 32 hours post fertilization a zebrafish embryo has a circulatory system and a fully functional innate immune system, along with a variety of distinct tissue types (Figure 1A), making it a self-contained ‘laboratory’ for the study of bacterial infection.

In this work we have investigated the earliest events in leptospirosis by inoculating the developing zebrafish with *L. interrogans* sv. Copenhageni. During the first 36 hours of infection, *L. interrogans* produces persistent infection in the zebrafish embryo, with phagocytes playing a central role in the initial host response to infection and possibly in the localization of leptospires to target tissues.

## Materials and Methods

### Animal care and strains

Wild-type AB zebrafish embryos were maintained and infected by injection into the caudal vein or hindbrain ventricle as described previously [22],[23],[26] at 24–30 hours post fertilization unless otherwise noted [22],[23],[26]. All animals were handled in strict accordance with good animal practice as defined by the relevant national and/or local animal welfare bodies, and all animal work was approved by the University of Washington Institutional Animal Care and Use Committee.

### Bacterial strains

*Leptospira interrogans* serovar Copenhageni strain Fiocruz L1-130 was isolated from a patient in Salvador, Brazil [27]. Virulent leptospires isolated from infected Golden Syrian hamsters were grown in EMJH medium supplemented with 1% rabbit serum and 100 ug/mL 5-fluorouracil at 30°C [28]. Staining was performed in a 1∶1000 dilution of SYTO-83 (Invitrogen) for 30 minutes, followed by rinsing with PBS to remove unbound dye. Inoculum was estimated based on fluorescence microscopy after injection.

### Microscopy

Widefield microscopy was performed on a Nikon E600 compound microscope equipped with DIC optics and 100 W Mercury lamp for epifluorescence. Objectives used included 10× Plan Fluor, 0.3 NA, 20× Plan Fluor, 0.5 NA, and 60× Water Fluor, 1.0 NA. Widefield fluorescence and DIC images were captured on a CoolSnap CF CCD camera (Photometrics) using MetaMorph 7.1 (Molecular Devices).

### Image processing

Dataset analysis and visualization was performed using MetaMorph 7.1 (Molecular Devices). Movies were produced from stacks compiled in MetaMorph. Additional movie compilation and formatting was performed in Adobe Premiere 6.0 and QuickTimePro 7.4 (Apple). Figure processing and assembly were performed in Adobe Photoshop CS2.

## Results

To determine the effect of *Leptospira interrogans* infection on developing zebrafish, we injected doses of roughly 10 to 100 organisms into 30 hour post fertilization zebrafish by either the caudal vein or hindbrain ventricle (Figure 1A). Inoculation by either route resulted in no lethality or gross pathology over 48-hours although organisms were detectable by DIC microscopy as long as 24 hours post-infection. Extracellular organisms were observed immediately after injection (Figure 1B) and video microscopy revealed that they exhibited the flexing, bending and spinning motility characteristic of these organisms in vitro (Video S1). These organisms appeared to be phagocytosed rapidly; within the first two hours post intravenous infection we found many macrophages in the blood contained leptospira. To ascertain that we were observing intracellular Leptospira by DIC microscopy, we stained *L. interrogans* cultures with SYTO 83 to render them red fluorescent before injection, and visualized infection with both DIC and fluorescence microscopy. Again by four hpi we were unable to detect extracellular bacteria at any location in the zebrafish embryos and all organisms were visualized within phagocytes, presumably macrophages based upon their morphology (Figure 1C). While the SYTO 83 stain confirmed the rapid intracellular localization of the organisms, we found that it diminished motility of stained bacteria in vitro. reducing motility from 100% immediately after staining to 10% at six hours post staining. Therefore, we performed all subsequent experiments using both stained and unstained bacteria to ensure that the observed infection phenotypes were not simply an artifact of bacterial compromise due to staining.

To examine the capacity of leptospires to attract macrophages, we injected similar doses of *L. interrogans* into the hindbrain ventricle at 30 hours post infection, a time in development when very few if any macrophages reside in this compartment [29]. Macrophages were rapidly recruited to the ventricle and took up the bacteria within the first four hours (Figure 1D). This result showed that the uptake of leptospires by macrophages did not require blood flow to bring the two together, and that macrophages actively migrated to the site of infection.

After encountering and taking up leptospires in the bloodstream or the hindbrain ventricle, the macrophages that contained organisms took on a distinct morphology. Although the bacteria appeared to be contained within compartments separate from the cytoplasm (Figure 1B, D), the macrophages generated numerous small vesicles which moved rapidly about the cytoplasm (Figure 1E and Video S2). Occasional membrane blebbing was also visible (Video S3). Again we note that both stained and unstained leptospira produced similarly unaffected embryos with the same characteristic-looking phagocytes during the first day of infection.

**Figure 2:**
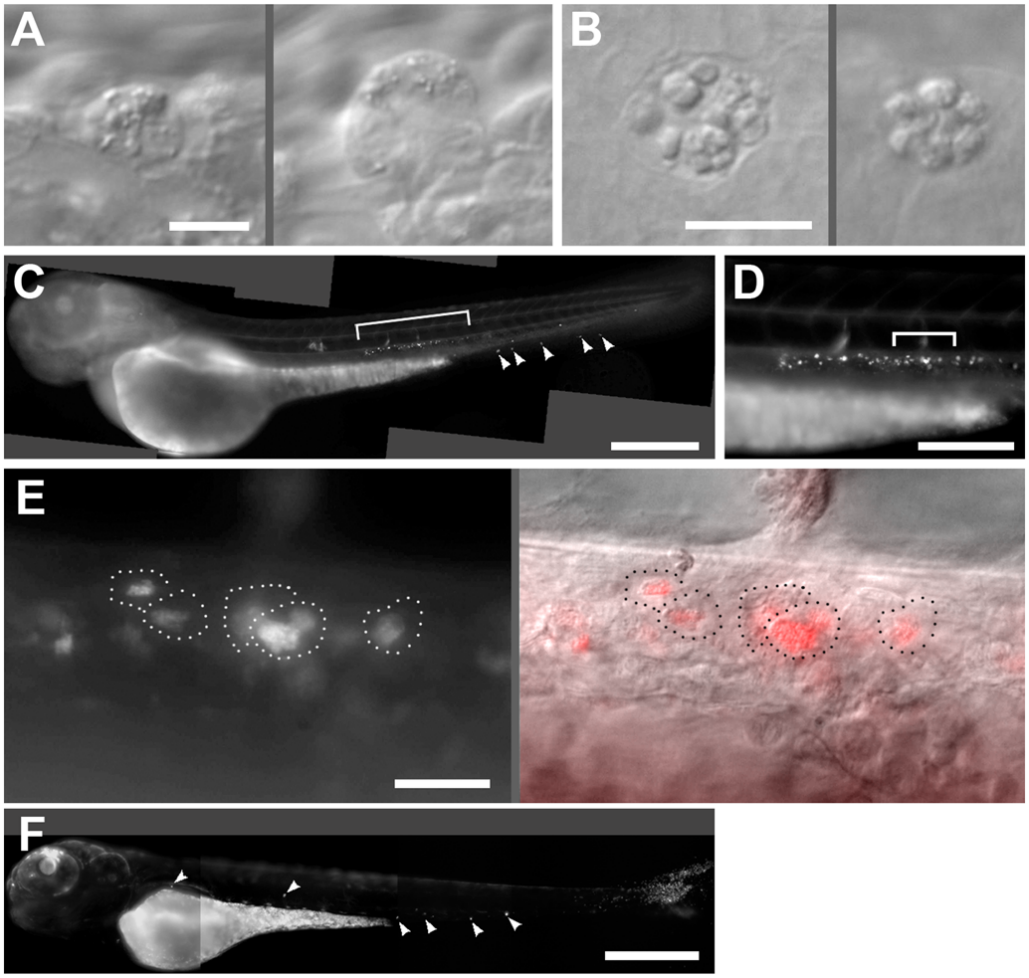
Leptospirosis of the zebrafish embryo at 24 hours post infection. A. Two affected cells in the caudal vein containing cytoplamsic vesicles, now larger. This embryo was infected intavenously. Scale bar, 10 µm. B. Affected cells in the brain, apparently containing clusters of undigested apoptotic bodies. This embryo was infected via hindbrain ventricle. Scale bar, 10 µm. C. Fluorescent image of whole embryo infected intravenously with SYTO 83-stained leptospira. While some fluorescent leptospires appear around the ventral tail (arrowheads), the majority have localized near the dorsal aorta (bracket). Scale bar, 300 µm. D. Higher magnification of the area bracketed in E, showing numerous distinct clusters of stained leptospires lateral to the dorsal aorta, just ventral to the notochord. Scale bar, 100 µm. E. Higher magnification of the area bracketed in D, with SYTO 83 fluorescence to the left and DIC overlay to the right. See Video S5. Dotted lines indicate the outlines of infected cells. Scale bar 20 µm. F. Fluorescence image of embryo 24 hours after infection with green fluorescent *P. aeruginosa*. Infected cells (arrowheads) appear in various places throughout the circulation. Scale bar 300 µm.

By 24 hours post infection, there was no gross pathology although bacteria were still plentiful. In experiments where fluorescent bacteria were used, all fluorescence correlated with intracellular clusters, always found in cells of a similar phenotype as the day before—many subcellular vesicles were present, often moving throughout the cytoplasm (Figure 2A, Video S4). Cells of similar morphology were found in embryos infected with unstained leptospires. The affected cells found in the brain also contained several larger vesicles consistent with apoptotic bodies (Figure 2B). Such cells were common in embryos infected via hindbrain, found occasionally in embryos infected intravenously, but not in uninfected controls (data not shown).

The most striking feature of embryos 24 hours after intravenous infection was the localization of fluorescent bacteria. While some fluorescent clusters were present in the caudal vein (Figure 2C, arrowheads), the majority were dorsolateral to the dorsal aorta in the trunk (Figure 2D–E, Video S5), a location which has been shown to play a part in early hematopoiesis [30]. Because of the location deep within the tissues, it was not possible to verify that the same localization occurred after infection with unstained bacteria. Previous experimental infections of zebrafish embryos with other organisms have not demonstrated such localization, suggesting that this accumulation is specific to infection with *L. interrogans*. To confirm this suggestion, we compared leptospiral infection to infection with fluorescent *Pseudomonas aeruginosa* over the same time course. Infection with *P. aeruginosa* produces either overwhelming infection or clearance over the first 36 hours of infection, depending upon dosage [25]. At 24 hours post infection with a non-lethal dose of *P. aeruginosa*, we found that the remaining bacteria were similar in number to *L. interrogans* remaining at 24 hours. Despite the fact that both bacterial types were apparently contained within phagocytes at this time, there was no accumulation of *P. aeruginosa*-infected cells in the region dorsolateral to the dorsal aorta (Figure 2F). While this phenomenon could represent a general mechanism whereby dead or compromised organisms are transported to this location, we note that we have not seen such a localization with heat-killed fluorescent *M. marinum*. Therefore, we suspect that this localization is the result of specific host-bacterial interaction.

## Discussion

We undertook the study of leptospiral infection of zebrafish embryos to assess the usefulness of zebrafish as a general model host for infection, as well as to examine the details of early pathogenesis directly in vivo. While *Borrelia burgdorferi*, another spirochete, has been observed in vivo during early pathogenesis [31],[32], this is the first in vivo visualization of leptospirosis of which we are aware. At least in embryos, infection of zebrafish with *L. interrogans* appears to be asymptomatic for the first 48 hours. It is not clear if this trend is inherent to the host-pathogen interaction or perhaps due to the lack of a functional adaptive immune system at this point in zebrafish development [33]. Also, it is possible that more damaging effects of infection require more than 48 hours to develop. At any rate, the immediate response of zebrafish embryos to *L. interrogans* infection is as follows. In contrast to *B. burgdorferi* in mice, which directly migrate out of the vasculature [31], Leptospires are taken up by phagocytes within a few hours of injection. The lack of antibody of any kind at this early stage in development demonstrates that it is not required for phagocytosis, as has been suggested [9],[34],[35]. The zebrafish complement system appears to be quite functional by this time [36], so it may be that complement-based opsonization is all that is required. Phagocytosis does not appear to rely upon accidental encounters with phagocytes, but may instead involve chemotactic mechanisms, as injection into the hindbrain, which normally contains very few if any phagocytes [29], results in active migration of macrophages to the site of infection. Proposed models for leptospiral pathogenesis mostly describe extended periods of leptospiremia, with extracellular bacteria finding their way into target tissues [4],[37],[38]. Defense against extracellular organisms, particularly in the early phases of infection, is likely to involve the innate defense mechanism of complement-based opsonophagocytosis. Supporting the relevance of the observations we report here, intracellular leptospires have been observed within splenic phagocytes by immunohistochemistry in the hamster model of leptospirosis [39]. It is possible that the infecting dose used in our experiments was too low to simulate a pathogenic infection. Barring this, however, our results suggest that leptospires are intracellular from very early in infection.

Observations of zebrafish embryonic macrophages that have ingested leptospires suggest that this phagocytosis may result in adverse cellular events. The appearance of small to medium sized vesicles moving about the cytoplasm takes place within one or two hours of the encounter, and infected cells with this characteristic morphology are still visible 24 hours later. It is not certain if this represents the persistence of the same affected cells, gradually gaining more vesicles, or the death of the initial macrophage followed by re-uptake of bacteria by another cell. Indeed, there has been evidence of apoptotic effects on infected host cells [40],[41],[42],[43], and we report here the blebbing appearance of affected cells after hindbrain infection. In our observations this blebbing was relatively rare, and so further observations are required to learn how relevant it is to pathogenesis.

It has been shown that some of the macrophages within the yolk circulation valley at the advent of circulation actually migrate into the brain, change their gene expression profiles, and become microglia [29]. These cells then collect and dispose of apoptotic bodies of neurons [29],[44], although they are also capable of fighting infection [22]. By 24 hours after injection of *L. interrogans* into the hindbrain ventricle, these cells are often seen to contain clusters of multiple apoptotic bodies, strikingly similar to microglia made incapable of digesting their cargo by knockdown of v0-ATPase a1 [44]. To our knowledge, functional impairment of macrophages after leptospiral infection has never been reported. When combined with the experimental approaches for detection and perturbation of phagolysosome fusion of Peri et al [44], the zebrafish model provides an ideal opportunity to explore the mechanisms of leptospiral effects on macrophages.

At 24 hours post infection, leptospira were conspicuously located dorsolateral to the dorsal aorta. This location corresponds to that of early hematopoietic cells populating a tissue analogous to the ‘aorta-gonad-mesonephros’ (AGM) hematopoietic tissues in developing mammals [30]. Blood cell precursors migrate from this area to the caudal hematopoietic tissue (CHT) in the ventral tail, starting around 24 hours post fertilization. While some infected cells were indeed found in the CHT (Figure 2C), there were consistently more at or near the AGM. It should be noted that due to technical limits of DIC microscopy, this localization was noted only when injecting leptospires stained with SYTO 83, which impairs bacterial motility. Since all earlier features of the infection appear to be unaffected by the stain, however, we consider it likely that this localization is not an artifact of staining but this will need to be verified with intrinsically fluorescent strains. The developmental timing of our observation of infected cells here corresponds with the later times of AGM to CHT migration (which ends around 72 hours post fertilization) [30], so the trunk tissue could still be acting as a hematopoietic site. The fate of this tissue, after its period as a hematopoietic zone, is unknown, and from our studies it is not clear whether the infection is within cells destined to depart or within other more permanent cells. The strikingly specific delivery of leptospires to this tissue by phagocytes provides insights into pathogenesis by suggesting a novel mechanism for targeting of organs during leptospiral dissemination.

## Supporting Information

Video S1 A single leptospire in the hindbrain ventricle shortly after injection.
(1.69 MB MOV)

Video S2 Phagocytes with intracellular vesicles, shortly after intravenous infection.
(1.97 MB MOV)

Video S3 Infected cells in the hindbrain ventricle. The lowermost cell undergoes blebbing similar to that seen during apoptosis.
(1.49 MB MOV)

Video S4 Phagocytes in the blood flow with large cytoplasmic vesicles at 24 hours post infection.
(1.80 MB MOV)

Video S5 Cells containing leptospires at 24 hours post infection, dorsolateral to the dorsal aorta.
(1.73 MB MOV)
